# Biochar rebuilds the network complexity of rare and abundant microbial taxa in reclaimed soil of mining areas to cooperatively avert cadmium stress

**DOI:** 10.3389/fmicb.2022.972300

**Published:** 2022-08-02

**Authors:** Yanfeng Zhu, Xiaoping Ge, Liping Wang, Yunnan You, Yanjun Cheng, Jing Ma, Fu Chen

**Affiliations:** ^1^Engineering Research Center of Ministry of Education for Mine Ecological Restoration, Xuzhou, China; ^2^School of Environment and Spatial Informatics, China University of Mining and Technology, Xuzhou, China; ^3^College of Hydrology and Water Resources, Hohai University, Nanjing, China; ^4^School of Public Administration, Hohai University, Nanjing, China

**Keywords:** biochar, mine reclamation, Cd-contaminated soil, rare and abundant microbial taxa, microbial network

## Abstract

Understanding the interactions between the soil microbial communities and species is critical in the remediation of heavy metal-contaminated soil. Biochar has been widely applied as a stabilizer in the *in situ* remediation of cadmium (Cd)-contaminated soils in mining areas. However, the rebuilding of the microbial taxa of rare and abundant species by biochar and their cooperative resistance to Cd stress remains elusive. In this pursuit, the present study envisaged the effects of two types of biochars *viz*., poplar bark biochar (PB) and thiourea-modified poplar bark biochar (TP) on the rare and abundant bacterial and fungal taxa by using pot experiments. The results demonstrated that the PB and TP treatments significantly reduced the leached Cd content, by 35.13 and 68.05%, respectively, compared with the control group (CK), in the reclaimed soil of the mining area. The application of biochar significantly improved the physicochemical properties like pH and Soil Organic Matter (SOM) of the soil. It was observed that TP treatment was superior to the PB and CK groups in increasing the diversity of the soil abundant and rare species of microbial taxa. Compared with the CK group, the application of PB and TP enhanced and elevated the complexity of the microbial networks of rare and abundant taxa, increased the number and types of network core microorganisms, reshaped the network core microorganisms and hubs, and boosted the microbial resistance to Cd stress. Our results indicate the response of rare and abundant microbial taxa to biochar application and the mechanism of their synergistic remediation of Cd-contaminated soil, thereby providing technical feasibility for *in situ* remediation of Cd-contaminated soil in mining areas.

## Introduction

Mining and industrial activities increase the risk of soil heavy metal contamination, especially by cadmium (Cd) (Huang et al., 2018; Kan et al., 2021). Cd is a highly toxic non-essential metal that is listed as the most hazardous heavy metal element by the United Nations (Zeng et al., 2019). Its long residence time in soil and high exposure risk to humans make it one of the priority pollutants (Xue et al., 2022). Mining activities often lead to serious surface damage and environmental pollution (Ma et al., 2021). Besides, the conventional mine reclamation adopts coal gangue and fly ash filling technology, which easily causes the leaching of harmful heavy metals, further aggravating the heavy metal pollution of the reclaimed mine (Dong et al., 2016). Once these lands are converted to agricultural land, they pose a great threat to food security, ecosystems, and human health (Lu et al., 2021; Zhu et al., 2022a). Therefore, there is an urgent need of an *in situ*, low-cost, and efficient remediation technology to solve the problem of Cd pollution in the reclaimed mines.

Compared with the physical and bioremediation strategies such as electrokinetic remediation, phytoremediation, and microbial remediation, the *in situ* stabilizer remediation has been widely applied in Cd-contaminated soil remediation, owing to the high efficiency and cost-effectiveness of the stabilizers in reducing the toxicity and bioavailability of Cd (Chen et al., 2018; Ma et al., 2020a,b). Since the stabilizers vary widely in performance, efficiency, and potential stabilization mechanisms, the selection and application method of the stabilizers plays a key role in the *in situ* remediation (Wang et al., 2021). Among the various Cd stabilizers, biochar has attracted great attention from scholars, owing to its multiple effects on remediation of Cd-contaminated soil, including reducing the bioavailability of Cd, alleviating soil acidification, and improving soil ecological functions (Ahmad et al., 2014; Chen et al., 2020). For a better stabilization effect, some studies have introduced exogenous elements in biochar (Gondek et al., 2018; Rajendran et al., 2019). These elements may combine with Cd in the soil to form more stable compounds which could be immobilized in the soil, achieving an efficient remediation.

Along with the efficient stabilization of Cd, extensive studies have focused and highlighted the ecological impact of biochar on the microbial taxa (Xu et al., 2022; Zhu et al., 2022c). In the natural environment, the abundance and distribution of species in microbial communities are non-uniform, with a few abundant species and a lot of rare species (Jiao and Lu, 2020). Different microbes play key roles in maintaining the ecosystem functions, including nutrient cycling, organic matter decomposition, soil health, and crop productivity (Jiao et al., 2018). However, various degrees of interference of Cd in soil may directly damage the normal physiological metabolism of the microorganisms, which may pose adverse effects on the microbial diversity along with the ecosystems (Wang et al., 2019). During soil remediation, abundant microbial taxa have drawn attention for their remarkable contributions to increase in the biomass and improvement of nutrient cycling (Qi et al., 2022). In the recent years, there is an increasing research on the importance of rare taxa in maintaining the ecosystem stability. Rare taxa have a high diversity and functional redundancy, and thus play an important role in ensuring the functions of the microbial communities (Hannula et al., 2017; Shu et al., 2021). Studies have revealed interactions of the intra-/inter-species between the rare and abundant species in resisting the disturbance due to the pollutants (Dong et al., 2021). They formed a complex ecological network and maintained the stability of the microbial network. Notably, some species, regardless of their amounts, occupied key positions in the ecological networks and were considered as key species for maintaining the stability of the community structure (Li et al., 2022). However, it is still unclear that whether these key species were abundant or rare, and their responses to environmental perturbations were not consistent all the time (Jiao and Lu, 2020; Zhao et al., 2022). For example, because of their poor resistance to heavy metals, almost all rare taxa in pristine soils were eliminated by heavy metals, resulting in a sharp decline in bacterial diversity. Studies have also shown that the diversity and community composition of rare taxa was more stable under the influence of climate change and other disturbances such as copper stress, freeze-thaw, and mechanical disturbance (Shade et al., 2014; Liang et al., 2020). During the *in situ* remediation of Cd-contaminated soil in mining areas, biochar changed the soil environmental conditions, including heavy metal concentrations, metal forms, soil pH, and available nutrients. These changes affected the aggregation and distribution of rare and abundant microbial taxa as well as their functions. Therefore, there is a need to identify the rare and abundant species and their interactions in the process of biochar remediation of Cd-contaminated soil.

In the present study, two high-efficiency Cd-stabilizing biochars *viz*., the poplar bark biochar (PB) and thiourea-modified poplar bark biochar (TP) were selected to conduct pot experiments for exploring the effects of biochar remediation in Cd-contaminated soil on the rare and abundant microbial taxa in mining areas and revealing their interactions and the mechanism of their synergistic control of Cd pollution with biochar. The specific goals of this study were as follows: (1) to elucidate the response of the rare and abundant taxa of bacteria and fungi to PB and TP application; (2) to explore the co-occurrence relationship of the rare and abundant taxa of bacteria and fungi under PB and TP application; and (3) to reveal the synergistic mechanism of biochar along with the rare and abundant microbial taxa against Cd stress. Our study can assist the prediction of the response of soil bacteria and fungi to biochar reclamation of Cd-contaminated soil in mining areas, and provide a technical support for further engineering application of biochar in the *in situ* remediation of heavy metal pollution in mining areas.

## Materials and methods

### Biochar preparation and soil sampling

In this study, the bark of Italian poplar was selected as the biochar material. The bark was fully washed with deionized water and dried in an oven at 60°C for 24 h. After dried, it was fully compacted and placed in a tube furnace which was under argon and with temperature raised to 600°C at 5°C/min and then kept at this temperature for 2 h. Therefore, PB was obtained. TP was prepared following the same procedure using bark and thiourea at a mass ratio of 1:1. The prepared biochar was ground, passed through a 60-mesh sieve and stored. The biochar produced by this method exhibited an optimal Cd adsorption efficiency, according to our earlier findings (Zhu et al., 2020a).

Soil samples (0–20 cm deep) were collected from the Liuxin Coal Mine Reclamation Area, Xuzhou City, Jiangsu Province, China. The area had a temperate continental monsoon climate, with an annual precipitation of 800–930 mm and an annual average temperature of 14.2°C. The type of soil was cinnamon soil, containing 29.19 ± 1.33% sand, 32.69 ± 1.02% clay, and 38.12 ± 1.58% silt. The cropping system was wheat-rice rotation. The Cd content in the soil was 9.97 ± 0.01 mg⋅kg^–1^. More details of the biochar and soil are shown in Supplementary Table 1.

### Pot experiment and soil physicochemical analysis

Pot experiments were performed in a temperature and humidity-controlled glass greenhouse. About 2.5 kg of soil was thoroughly mixed with biochar at a ratio of 2% (w/w) and filled into a polyethylene flowerpot with a height of 12 cm and a diameter of 13 cm. Our previous study confirmed that the best Cd stabilization effect was obtained at this biochar ratio (Zhu et al., 2020b). The treatment group without biochar was the control (CK) group, and the treatment group with poplar bark biochar and thiourea-modified biochar were the PB and TP treatment groups, respectively. A total of 10 replicas were set up for each treatment group. Cabbage seeds were sterilized with 2% hydrogen peroxide, washed with deionized water, and then sown at 2 cm depth for germination. Subsequently, the seeds were incubated at 25°C with 60% relative humidity for 30 days. Finally, 0–10 cm topsoil samples from three sampling points were collected from each pot and mixed together as the soil sample.

The soil pH and electrical conductivity (EC) was obtained by measuring a 1:2.5 (w/v) soil-water suspension using a pH meter (Fan et al., 2020). The soil organic matter (SOM) was determined by potassium dichromate oxidation-outer heating method (Luo Z. et al., 2020). NH_4_^+^-N was measured by spectrophotometry after extraction with potassium chloride, and NO_3_^–^-N was determined by spectrophotometry after extraction with calcium chloride (Li et al., 2020). The available phosphate (AP) was determined by molybdenum blue method after extraction with ammonium bicarbonate (Zhu et al., 2022b). The enzymatic activities of β-glucosidase (BG), alkaline phosphatase (Pho), and urease (Ure) were determined by nitrophenol colorimetry, disodium phenyl phosphate colorimetry, and sodium phenoxide-sodium hypochlorite colorimetry, respectively (Zhu et al., 2022b). After the soil was digested with HNO_3_^–^-HF, the total Cd content in the soil was analyzed by inductively coupled plasma mass spectrometer (ICP-MS) (Zhao et al., 2019). The four-step sequential extraction method proposed by the European Community Bureau of Reference (BCR) was adopted to determine the forms of Cd in soil samples, including the weak acid-soluble state, reducible state, oxidizable state, and residue state (Xu et al., 2020). The content of Cd in the leaching solution was determined according to the toxicity characteristic leaching procedure (TCLP) (Luo M. et al., 2020). The relative standard deviation (RSD) among replicate samples was smaller than 10%.

### DNA extraction, PCR and high-throughput sequencing

DNA was extracted from soil samples using the FastDNA™ SPIN Kit for soil (MP Biomedicals, California, CA, United States) according to manufacturer’s instructions. The V4-V5 region of 16S rRNA was amplified using primers 338F (5′-ACTCCTACGGGAGGCAGCAG-3′) and 806R (5′-GGACTACHVGGGTWTCTAAT-3′). The PCR program was as follows: 95°C for 3 min, followed by 27 cycles of (95°C for 30 s, 55°C for 30 s, and 72°C for 30 s), and 72°C for 10 min and finally cooled to 10°C. The PCR products of the various samples were detected by 2% agarose gel electrophoresis. A DNA Gel Extraction Kit (Axygen, United States) was used to recover the target PCR fragments. According to the above preliminarily quantitatively determined DNA amount from electrophoresis results, a Quant-iT™ PicoGreen™ dsDNA Assay Kit with fluorescent reagents was used to conduct fluorescence quantification on PCR amplification products using a microplate reader (BioTek, FLx800, United States). The samples were mixed in equal proportions. The TruSeq Nano DNA LT Library Prep Kit developed by Illumina (United States) was used to construct the DNA sequencing library. The constructed library was quantified by Qubit and qPCR. The qualified library was sent to the Shanghai Meiji Biotechnology Co., Ltd., for sequencing with HiSeq 2500 PE2500 (Illumina, United States) (Li et al., 2020; Luo Z. et al., 2020).

A program called Trimmomatic (Version 0.33^1^) was used to filter the poor quality paired-end (PE) raw reads. FLASH (Version 1.2.11^2^) was used to merge the PE rends into one sequence. According to the barcode and primer information at the two ends of each sequence, Mothur (Version 1.35.1^3^) was used to assign the sequence to the corresponding sample, and finally an effective assembled sequence was obtained. USEARCH was adopted to cluster the assembled sequences (Version 10^4^), and the sequences were clustered into amplicon sequence variants (ASVs) with a sequence similarity of 97%. USEARCH was also run to remove chimera and singleton sequences. Then Silva^5^ was carried out to annotate the representative sequences (Ma et al., 2021).

### Identification of rare and abundant microbial taxa

To assess the response of the rare and abundant microbial taxa to the biochar treatments, the ASVs were classified into the following six categories based on the criteria used in Chen’s study (Chen et al., 2019): (i) always abundant taxa (AAT), ASV with a relatively abundance of ≥1% in all samples; (ii) conditionally abundant taxa (CAT), ASV with a relatively abundance of >1% in some samples but never <0.01%; (iii) always rare taxa (ART), ASV with a relatively abundance of <0.01% in all samples; (iv) conditionally rare taxa (CRT), ASV with a relative abundance of <1% in all samples and <0.01% in some samples; (v) moderate taxa (MT), ASV with a relative abundance between 0.01 and 1% in all samples; (vi) conditionally rare and abundant taxa (CRAT), ASV with a relative abundance ranging from <0.01 to ≥1%. According to previous studies, AAT, CAT, and CRAT were combined as abundant taxa, while ART and CRT were combined as rare taxa.

### Data process and analysis

The α-diversity indices of the rare and abundant microorganisms were obtained by the vegan package in R (Version 4.1.3). One-way analysis of variance (ANOVA) was adopted to test the significance of differences between the treatments, and the results were tested by honestly significant difference (HSD) test. Based on the Bray-Curtis dissimilarity matrix, the β-diversity of microorganisms was further determined. Subsequently, the differences in the different microbial communities were visualized by principal coordinate analysis (PCoA) with ggplot2 package. Permutational multivariate analysis of variance (PERMANOVA) was performed using the vegan package to elucidate the significant differences in the microbial subcommunity structure between the different groups. Using the pairwise Spearman rank-correlation matrix, co-occurrence networks were constructed with the help of the psych package. Robots (soft threshold *r* > 0.60) and statistically significant (*p* < 0.05) correlations were included in the network analysis. The visualization of co-occurrence networks was carried out in Gephi 0.9.2. Network topological characteristics including average degree, clustering coefficient and betweenness centrality were obtained by the igraph package. The network keystone ASVs were determined according to within-module connectivity (Zi) and among-module connectivity (Pi), and nodes with Zi ≥ 2.5 or Pi ≥ 0.62 were considered as the keystone ASVs. Mantel correlation was used to explore the relationship between the Bray-Curtis dissimilarity and Cd content of the rare and abundant microbial communities. Finally, the canonical correspondence analysis (CCA) was conducted using the vegan package to evaluate the impact of soil variables on the different microbial sub-communities.

## Results

### Changes in the reclaimed soil and plant properties after biochar application

**FIGURE 1:**
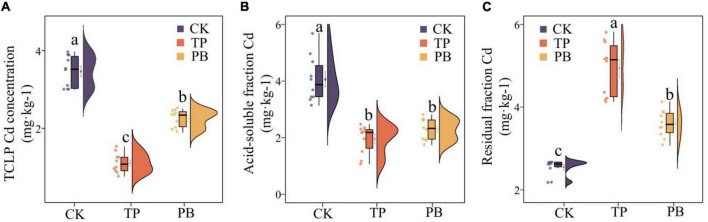
Leached cadmium (Cd) content **(A)** and distribution forms of Cd **(B,C)** in the control group (CK), poplar bark biochar (PB), and thiourea-modified poplar bark biochar (TP) soil treatment groups. Different letters indicate the values that differ significantly among CK, PB, and TP treatments at *p* < 0.05 [honestly significant difference (HSD) test].

The results showed that the effects of PB and TP on the physicochemical properties, activities of enzymes and Cd content of the soil in mining areas were significantly different (*p* < 0.05). Compared with the CK group, the leached Cd content in the PB and TP treatment groups was decreased by 35.13 and 68.05%, respectively, the acid-soluble Cd content decreased by 43.60 and 52.02%, respectively, and the residual Cd content was increased by 18.39 and 51.31%, respectively (Figure 1). Compared with the CK group, the PB treatment significantly increased the SOM and AP, while the TP treatment significantly increased pH, EC, and AP. The application of biochar showed nearly no effect on the soil ammonium nitrogen content, but they significantly dropped the soil nitrate nitrogen content (Supplementary Table 2). Compared with CK, PB, and TP significantly promoted crop growth (*p* < 0.05), plant height increased from 10.1 to 12.1 cm, and 13.4 cm, respectively, while leaf length increased from 5.59 to 6.09 cm, and 6.85 cm, respectively. Meanwhile, biomass of roots, stems, and leaves of PB treatment increased 23.91, 30.58, and 9.97%, as well as the TP treatment increasing 83.88, 97.15, and 21.80%, respectively (Supplementary Figure 1). Compared with the CK group, the PB, and TP treatments had no significant effects on the soil alkaline phosphatase activity, but they significantly increased the contents of urease. Compared with the CK group, the PB treatment significantly decreased the enzymatic activity of β-glucosidase, whereas the TP treatment increased it (Supplementary Table 2).

### Distribution and diversity of the rare and abundant taxa in response to biochar application

There were 1,620,057 and 1,642,124 high-quality sequences of bacteria and fungi, respectively, in all samples after the quality control step, resulting 5,393 bacterial and 1,344 fungal ASVs, with a 97% sequence similarity. In case of bacteria, there were 956 rare taxa and 61 abundant taxa, while in case of fungi, 247 ASVs exhibited the rare taxa, and only 78 ASVs were classified as abundant taxa. The relative abundance of rare taxa was higher than that of abundant taxa. The soil samples were identified and classified into 39 bacterial phyla (Supplementary Figure 2). The top nine phyla with higher abundance accounted for more than 95% of the relative abundance. *Proteobacteria* is the most dominant phylum in all treatments, and the dominant phylum of abundant and rare bacteria is *Proteobacteria*, when its relative abundances of PB and TP treatments increased 30.47 and 93.46%, respectively, compared to CK group. The abundant *Sordariomycrtes* is dominant phyla of abundant fungi, while plylum *Dothideomycetes* belongs to the rare fungi.

**FIGURE 2:**
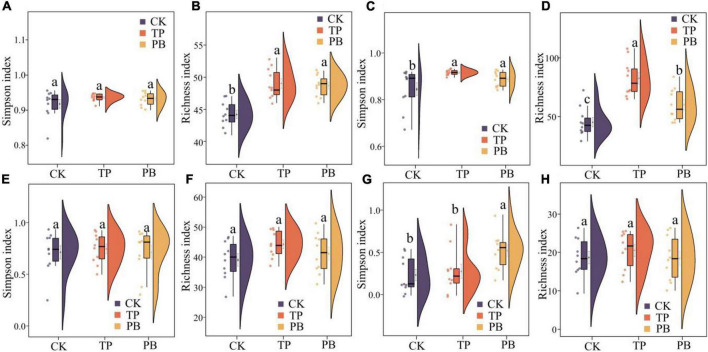
α-Diversity indices of abundant **(A,B)** and rare **(C,D)** bacterial and abundant **(E,F)** and rare **(G,H)** fungal communities in the control group (CK), poplar bark biochar (PB), and thiourea-modified poplar bark biochar (TP) treatment groups. Different letters indicate the values that differ significantly among CK, PB, and TP treatments at *p* < 0.05 [honestly significant difference (HSD) test].

**FIGURE 3:**
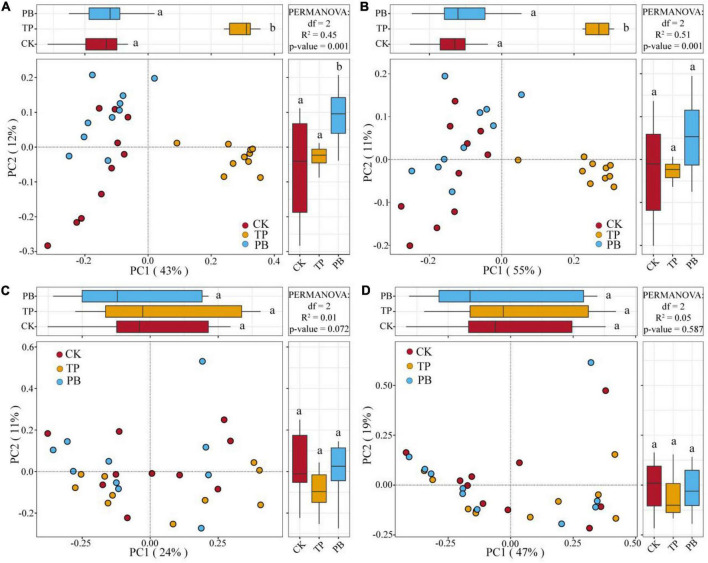
Principal coordinate analysis (PCoA) of the abundant and rare bacterial and fungal communities using Bray-Curtis distances. Different letters indicate the distribution of different treatments of samples along the PC1 and PC2 axes at *p* < 0.05. Abundant bacteria **(A)**, Rare bacteria **(B)**, Abundant fungi **(C)**, and rare fungi **(D)**.

The application of PB and TP did not change the Simpson index of abundant bacterial and fungal taxa, but both significantly increased the Simpson index of rare taxa (*p* < 0.05). The application of biochar significantly increased the richness of the rare bacterial and fungal taxa (*p* < 0.05), and no significant differences were seen in the richness of the abundant fungal taxa between the treatment groups. However, the PB and TP treatments significantly increased the richness of the abundant fungal taxa (*p* < 0.05; Figure 2). The results of PCoA analysis at the level of ASV exhibited a close distance between the PB and the CK group, indicating similar soil abundant and rare bacterial community structures between these two groups. The TP treatment resulted in very different abundant and rare bacterial community composition in the soil from those of the CK and PB treatments. The TP treatment showed a significant effect on the community composition of the rare and abundant bacteria (*p* = 0.001) (Figure 3). The similarity of rare and abundant fungal communities was less affected by biochar than that of the bacteria. Both the rare bacterial and fungal communities were less similar than their corresponding abundant communities, suggesting that the β-diversity of rare taxa was more susceptible to biochar application.

### Co-occurrence networks of the rare and abundant microbial taxa in response to biochar application

To further explore the response of interactions between the microbial taxa with various abundances in biochar application, co-occurrence networks of rare and abundant microbial communities were constructed at the ASV level based on Spearman correlation. All the co-occurrence networks exhibited scale-free characteristics (*R*^2^ > 0.835) (Supplementary Table 3), thereby indicating the non-random structures of the networks. In this study, the dominant modules in the networks were mostly composed of rare taxa. The rare nodes appeared at the edges in most of the abundant nodes.

**FIGURE 4:**
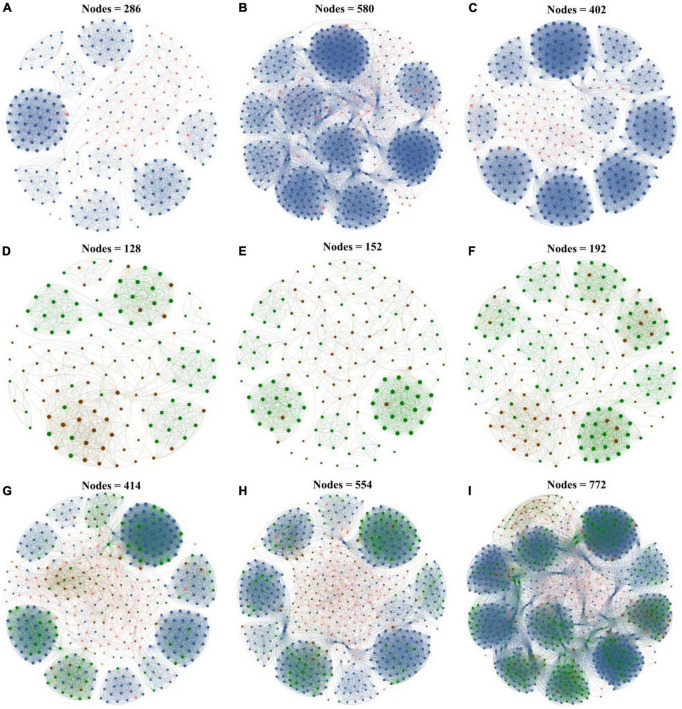
Microbial networks of the control group (CK), poplar bark biochar (PB), and thiourea-modified poplar bark biochar (TP) groups. **(A–C)** Rare and abundant bacterial networks; **(D–F)** rare and abundant fungal networks; and **(G–I)** rare and abundant bacterial and fungal networks. The size of a node is proportional to the degree of connectivity. Blue nodes: rare bacteria; pink nodes: abundant bacteria; green nodes: rare fungi; and brown nodes: abundant fungi.

In the bacterial networks of the CK, PB, and TP groups, the rare and abundant ASVs accounted for 79.02 and 20.98%, 90.00 and 10.00%, and 85.82 and 14.18% of the total nodes in the corresponding network, respectively (Figure 4), and 2 (both rare), 8 (all rare), and 6 (2 abundant and 4 rare) keystone ASVs were identified, respectively (Supplementary Figure 3). In the fungal networks of the CK, PB, and TP groups, the rare and abundant ASVs accounted for respective 53.12 and 46.88%, 52.63 and 47.37%, and 64.06 and 35.94% of the total nodes in the corresponding network, and 3 (2 abundant and 1 rare), 5 (4 abundant and 1 rare), and 5 (2 abundant and 3 rare) keystone ASVs were identified, respectively. The rare bacterial and fungal nodes in the CK, PB, and TP groups accounted for 54.59 and 16.43%, 62.27 and 14.44%, and 67.62 and 15.93% of the total nodes in the corresponding network, respectively. In the bacterial-fungal co-occurrence networks of the CK, PB, and TP groups, 13, 10, and 16 keystone ASVs were identified, with the abundant fungi accounting for 8/13, 7/10, and 1/2, respectively. The rare microbial taxa in the sub-network were the main body of the network, indicating that the taxonomic disappearance of these taxa may lead to the disintegration of the network and module. Thus, the rare taxa could be more important than the abundant taxa in maintaining the complexity of the microbial networks.

### Relationships between biochar, microbial communities, microbial networks, and cadmium stress

As the Cd concentration decreased, the bacterial and fungal co-occurrence networks became more complex, with an increased number of nodes and connections. Especially in the TP treatment group, the number of nodes of the rare bacterial and fungal taxa was higher than that compared with those of the CK and PB groups (Supplementary Table 3). The three important node-level topological characteristics of the subcommunities, degree, betweenness, and clustering coefficient showed that the betweenness of the TP group was significantly higher than that of CK and PB. A node exhibiting a high betweenness has a stronger control over the network. Therefore, the rare bacterial and fungal taxa may convey more information. The clustering coefficient of the TP group was significantly higher than that of the CK and PB, indicating that the interconnections between the adjacent points of microbial taxa in the TP group were higher. The results suggested that biochar affected the connections between the microbial communities, thereby increasing the complexity of the soil microbial community networks.

**FIGURE 5:**
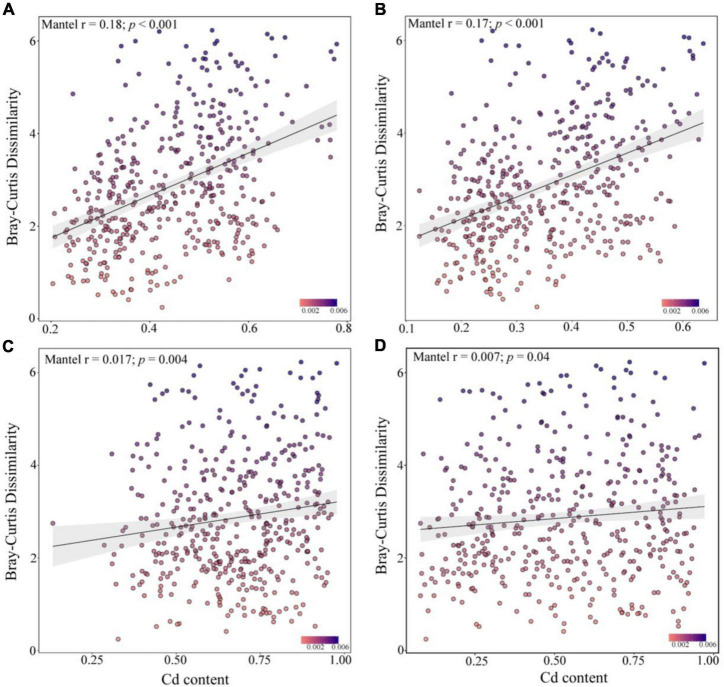
Mantel correlation between the dissimilarity of abundant bacteria **(A)**, rare bacteria **(B)**, abundant fungi **(C)**, and rare fungi **(D)** communities and the soil cadmium (Cd) levels.

Spearman’s correlation analysis showed that the community similarity between the rare and abundant bacterial taxa was significantly positively correlated with the leached Cd, acid-soluble Cd, oxidized Cd, reduced Cd, and residual Cd in the soil (*p* < 0.001, Figure 5). Soil Cd positively correlated with the community similarity of abundant fungal taxa, whereas the community similarity of the rare fungal taxa did not exhibit a significant correlation with Cd. Although the *R*^2^ of the correlation analysis was small, the Mantel test demonstrated that the compositions of the rare bacterial and abundant fungal communities presented higher correlations with various Cd forms in the soil than the compositions of the abundant bacterial and rare fungal communities. These results suggest that the changes in the rare and abundant microbial taxa may modulate the soil Cd levels after biochar application.

**FIGURE 6:**
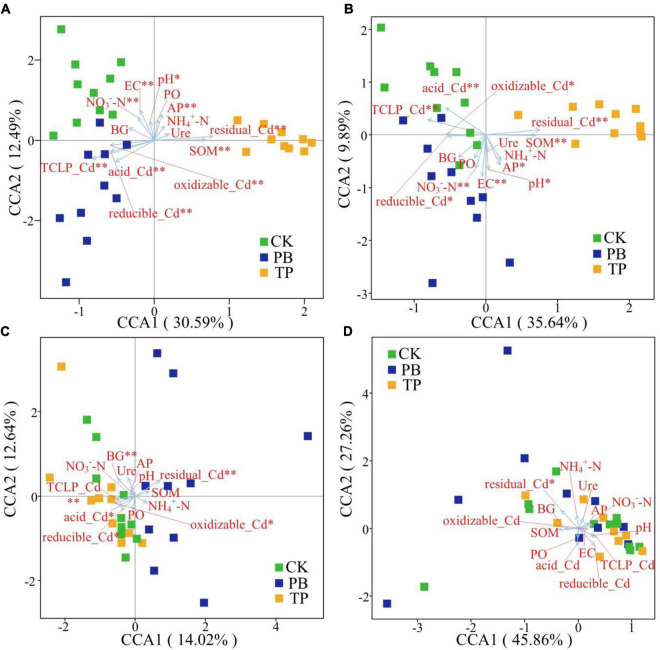
Identification of driving factors of β-diversity of the rare and abundant microbial communities in soil by Bray-Curtis distance-based canonical correspondence analysis (CCA). EC, electrical conductivity; SOM, soil organic matter; AP, available phosphorus; NO_3_^–^-N, nitrate nitrogen; NH_4_^+^-N, ammonical nitrogen; BG, β-glucosidase; Ure, urease; PO, alkaline phosphatase. *Indicates statistically significant. *p < 0.05; ^**^p < 0.01. Abundant bacteria **(A)**, Rare bacteria **(B)**, Abundant fungi **(C)**, and rare fungi **(D)**.

The Bray-Curtis distance-based CCA results showed that the rare and abundant bacterial taxa formed distinct and well-separated clusters in different treatment groups (Figure 6). However, the difference between the rare and abundant fungal taxa was not clear, thereby indicating that the changes in the soil properties after biochar treatment had a big difference in the impact of the rare and abundant taxa between the bacterial and fungal sub-communities. The impact on bacterial taxa was greater than that on fungi. The rare and abundant bacterial communities were mainly driven by pH, EC, SOM, AP, available nitrogen (AN), leached Cd content, and Cd forms. For fungi, BG, leached Cd content, and various forms of Cd were significantly associated with the changes in the abundant taxa. In the figure, the length of the arrow shows that the factor that showed the greatest influence on the number of soil microbial ASVs was the residual Cd. In addition, the sample points of the PB and TP treatments formed obtuse angles with the leached Cd, but acute angles with the residual Cd, indicating that the biochar treatment could help to reduce the leached Cd content and increase the residual Cd content.

## Discussion

The application of biochar could decrease the soil Cd availability, which might lead to changes of the external environmental stress (Zhu et al., 2022b). In this study, OH^–^ ions released from the surface of the biochar particles significantly increased soil pH (*p* < 0.05), from 7.87 to 7.92 and 7.97, respectively, with the PB and TP treatments (Supplementary Table 2). Compared with the CK group, the application of biochar increased the buffering capacity of the soil system. The fixation of soil Cd, including the adsorption, complexation, and precipitation of Cd ions increased in an alkaline environment, and this has been confirmed in previous studies (Ehsan et al., 2014; Hou et al., 2019; Li et al., 2019). The elevation in pH did not only increase the negative charge on the soil components to boost Cd fixation (Tang et al., 2020), but also initiated and enhanced the ability of other adsorption factors to adsorb Cd (Yang X. et al., 2016). For example, soil pH was the primary factor affecting the phosphorus fixation in soil, because it ensured sufficient adsorption sites for phosphate ions, when physical deposition occurs on the biochar surface (Jin et al., 2019). Besides, earlier studies have revealed that biochar improves the soil agglomeration structure, which reduces the transfer of water into the soil surface and enhanced the soil aeration (El-Naggar et al., 2018; Bashir et al., 2020). This promotes the growth of plant roots and microbes, thereby increasing the Cd adsorption by plant root exudates and microorganisms (Mazhar et al., 2019; Harindintwali et al., 2020). A large number of alkaline cations get adsorbed on the surface of the biochar and improve the conductivity of soil particles. While OH^–^ gets neutralized by the H^+^ ions absorbed by the clay minerals, the adsorption of Cd^2+^ occurs in the form of soil colloids (Ehsan et al., 2014). In addition, the sulfur element introduced by TP promotes the formation of cadmium sulfate, which cannot be easily oxidatively hydrolyzed and then greatly reduces the bioavailability of Cd (Gholami et al., 2019; Chen et al., 2020). It is generally found that adding biochar would decrease soil enzyme activity related to soil carbon mineralization (Zhu et al., 2022b). Bailey et al. (2011) have shown that the addition of biochar to soil has increased a series of enzymatic activities, and these enzymes were related with nitrogen utilization. The addition of biochar will inevitably change the soil carbon, nitrogen, and phosphorus nutrient cycle (Yang J. et al., 2016), and β-glucosidase, urease and alkaline phosphatase can be used as evaluation indicators. Elzobair et al. (2016) have proved that biochar had the ability to adsorb a variety of organic and inorganic molecules, as well as could inhibit several enzyme activities or enzyme substrates through adsorbing or blocking reaction sites. Moreover, the positive effects of biochar on soil enzymes (Ali et al., 2020) might have a relationship with the improvement of soil physicochemical properties. It is also worth noting that different additives showed the big divergence of effects on soil enzyme activity, which might be caused by the different constituent raw materials of these additives (Elzobair et al., 2016).

The adsorption of heavy metal ions on the biocarbon surface in the soil might change the soil C/N ratio, therefore, the addition of PB and TP have significantly increased the biomass of cabbage (Supplementary Figure 1). O’connor et al. (2018) have shown that high concentrations of Cd could pose potential negative effects on plant growth, whereas the addition of biochar could alleviate the fluidity and availability of heavy metals in soil. The macroscopic nutrients, for example, nitrogen, phosphorus, and alkaline cations (e.g., Ca^2+^ and Mg^2+^), could directly or indirectly increase the plant productivity through providing some nutrients or improving the soil structure (Chen et al., 2018). The cumulative absorption of Cd in plant tissues is always distributed in roots and shoots. However, most of these absorbed metals prefer to retain in the roots, while a limited portion would transfer to the aboveground parts. Plant iron carriers released from plant roots are important factors affecting the availability of trace elements in the rhizosphere, and this mechanism may protect plants from heavy metal poisoning and reduce metal transfer to plant tissues (Lu et al., 2019). Iron chelates are synthesized and secreted by grasses. Biochar is an important donor of iron oxides. These non-protein amino acids can dissolve a small amount of soluble iron compounds in the rhizosphere.

There were clear differences in the diversity and distribution of the rare and abundant taxa of bacterial and fungal communities caused by biochar. In terms of diversity, compared with the CK group without biochar application, the biochar treatments exhibited little effect on the Simpson indices of the abundant soil bacterial and fungal taxa, but significantly increased the Simpson index of the rare bacterial taxa (*p* < 0.05), thereby proving a superior effect of TP than that of PB (Figure 2). Interestingly, the diversity of the rare microbes was higher than that of the abundant microbes in all treatments. Because of the high diversity of the rare microbes, the rare microbial taxa may have increased the functional redundancy of the community, thereby providing a wider ecological buffer space to cope with Cd stress (Zhao et al., 2022). In terms of distribution, the rare and abundant bacterial taxa were clearly separated given biochar treatments, and the separation in the TP group was more distinct (Figure 3), thus indicating an intensified reconstructing of the bacterial taxa by the TP treatment. The distributions of the rare and abundant fungal communities in the treatment groups were quite different, and no clear clustering appeared. Biochar treatments may have little effect on the distribution and reconstructing the abundant and rare fungal taxa in the soil. Studies have shown that the rare microbial taxa can act as a microbial seed bank to safeguard the entire community (Dong et al., 2021; Xu et al., 2021). Our results demonstrated that the rare bacterial taxa (such as *cyanobacteria*) get activated by biochar, and thus maintained the bacterial community stability under pollution stress, which was not the case in the group with no biochar application. From the results of CCA analysis (Figure 6), it can be seen that the soil Cd was the primary factor affecting the distribution of the abundant and rare microbial communities in soil, especially the leached Cd content and residual Cd content in the soil. In addition, pH and SOM significantly affected the distribution of rare and abundant bacterial taxa. The applications of PB and TP increased the pH and buffering capacity of the soil, which further promoted the passivation of Cd and reduced its stress on microorganisms. The organic matter in biochar provided a nutrient-rich soil environment for microorganisms, especially TP, which greatly improved the soil nutrient status, thereby providing a good living environment for the abundant and rare microorganisms. After modification by biochar, the complexity of the microbial network increased, and the number and the types of core microorganisms was also elevated. The functional microorganisms such as *Proteobacteria* with pollutant degradation function (Banerjee et al., 2019) and *Actinomycetes* (Xu et al., 2017) with high metal resistance occupied the core positions of the network, maintaining the community stability under Cd stress.

Co-occurrence networks are an important means to explore the abundance patterns and internal relationships of the complex microbial communities (Kaya et al., 2020; Li et al., 2021). They display changes in the topology and characteristics of the rare and abundant microbial networks and serve to be a remarkable tool for the in-depth understanding of the stability of complex ecosystems. In the present study, the co-occurrence networks of microorganisms in all groups exhibited a power-law distribution, indicating that the networks were non-random and scale-free (Figure 4). Compared with the CK group, under the action of biochar in the PB and TP treatments, the abundant and rare taxa networks of bacteria and fungi were more complex, indicating that the contribution and tightness of the microbial networks were higher under the action of biochar. The frequent and diversified coupling relationship between the microorganisms provided a better buffer to the microorganisms to cope the Cd stress (Zhu et al., 2022b). Topological characteristics such as the degree of the nodes, betweenness, and closeness centrality suggested that the TP-modified microbial networks indicated a more complicated coupling between the rare and abundant taxa of bacteria and fungi. The reduction of TP in the leached Cd content and Cd acid-soluble state was higher than that in the PB and CK groups. The soil pH and available nutrients caused by TP created a good living environment to the soil microorganisms. Therefore, after the application TP, the complexity of the networks of the rare and abundant taxa increased, and the network structure was more organized. Furthermore, the application of biochar increased the number of core microorganisms in the network. In the bacterial networks, rare species of microorganisms accounted for a higher proportion of core microorganisms, such as *Proteobacteria, Actinobacteriota, Gemmatimonadota, Myxococcota, Chloroflexi, Patescibacteria, Bacteroidota*, and *Bdellovibrionota* (Supplementary Figure 1). Some of them were related to plant growth promotion and tolerance enhancement, such as *Gemmatimonadota* (Qiu et al., 2021), while some were associated with the metal-sensitivity, such as *Bacteroidota*. In the fungal networks, except for the TP treatment, most of the core microorganisms were abundant such as *Ascomycota* and *Basidiomycota* species. The phylum of *Ascomycota* was able to preferentially grow on the carbon-rich refractory materials and decompose cellulose, lignocellulose, and chitin in the soil. The main function of *Proteobacteria* is to promote the fixation of organic nitrogen and improve the adaptability to complex environments, which plays an important role in maintaining soil ecosystem functions. *Proteobacteria* is an abundant aerobic bacteria, which could degrade various pollutants and promote oxidative enzymes. Banerjee et al. (2019) have shown *Proteobacteria* have strong metabolic characteristics and environmental adaptability, and play an important role in immobilizing heavy metals and maintaining ecosystem functions. *Actinomycetes* have thicker peptidoglycan layers, which could provide high metal resistance (Xu et al., 2017). The phylum *Glomeromycota* has been reported that they could form a symbiotic relationship with plant roots, which bring about the increasing tolerance of plants to heavy metals (Lee et al., 2018). These microorganisms might be furtherly used to promote the stabilization of heavy metals in the soil, and enhance the fertility of the reclaimed soil, as well as speed up the agrochemical process of the soil in the mining area. It is worthy to mention that in the interaction networks between the bacteria and fungi, we found that most positions of the core microorganisms were occupied by fungi, while bacteria only occupied a small part. This indicated that under biochar modification, the fungal community can play a bigger role than bacteria in maintaining the stability of the microbial network, especially the abundant fungal community. The application of biochar can thus be beneficial to the formation of a more developed and healthy soil system, and it may serve as an important technical means to alleviate and solve the Cd stress in mining areas.

## Conclusion

Understanding the interactions between microbial taxa is very important for the reclamation of heavy metal polluted soils with biochar in mining areas. In the present study, the applications of PB and TP improved the physicochemical properties and enzymatic activities of the reclaimed soil in the mining area, and effectively reduced the soil Cd availability. Both PB and TP rebuilt the abundant and rare microbial communities in the Cd-contaminated soils. TP performed better in improving the diversity, structure and distribution patterns of the soil abundant and rare bacterial and fungal communities than PB. The network topology characteristics showed that the co-occurrence networks of bacteria and fungi modified by biochar exhibited a higher complexity and stability than that of CK, as well as increased the number and types of core microorganisms. The taxa that accounted for the majority of the core microorganisms in the bacterial and fungal networks, such as *Proteobacteria, Actinobacteria, Gemmatimonadota, Bacteroidota*, and *Basidiomycota* occupied the core hubs of the network and improved the resistance of the microbial communities to Cd stress. Our study can provide a technical support for the engineering application of biochar in the *in-situ* remediation of heavy metal pollution in mining areas.

## Data availability statement

The datasets presented in this study can be found in online repositories. The names of the repository/repositories and accession number(s) can be found in the article/Supplementary material.

## Author contributions

FC, YZ, and JM collected the samples. YZ, YY, and YC performed the experiments. YZ performed the data analyses and wrote the manuscript. FC, JM, XG, and LW helped to perform the analysis with constructive discussions. FC performed the supervision, project administration, and funding acquisition. All authors contributed to the revisions during the editing process and read and agreed to the published version of the manuscript.

## Footnotes

1
http://www.usadellab.org/

2
https://ccb.jhu.edu/software/FLASH/

3
http://www.mothur.org

4
http://www.drive5.com/usearch/

5
https://www.arb-silva.de/
