# Supplementary material for: Biochar rebuilds the network complexity of rare and abundant microbial taxa in reclaimed soil of mining areas to cooperatively avert cadmium stress
Source: Front Microbiol. 2022 Aug 2;13:972300. doi: 10.3389/fmicb.2022.972300 (PMC9378816; doi:10.3389/fmicb.2022.972300)
Supplement: Supplementary file 1 [file Data_Sheet_1.docx]

Supplementary Material

# Supplementary Data

All the figures (Figure 1-Figure 6) in the manuscript were saved in a zip file and uploaded as ‘Supplementary Material Presentation’ individually. The supplementary tables (Table S1-S3) and supplementary figures (Figure S1-S3) were placed in Section 2 of **Supplementary Material**. The data of bacteria-abundant ASVs, bacteria-bare ASVs, fungal-abundant ASVs and fungal-bare ASVs were uploaded as well.

# Supplementary Tables

# Table S1 Primary physicochemical properties of soil and biochar.

| Properties | Treatment | | |
| --- | --- | --- | --- |
|  | Experimental soil | PB | TP |
| pH | 6.86 ± 0.45c | 9.92 ± 0.21b | 10.85 ± 1.00a |
| Ash (%) | - | 41.68 ± 0.52b | 47.71 ± 0.29a |
| Surface area (m^2^·g^-1^) | - | 2.77 ± 0.18b | 5.70 ± 0.35a |
| Average pore diameter (nm) | - | 1.94 ± 0.03b | 3.42 ± 0.01a |
| EC (mS·cm^-3^) | 0.20 ± 0.25a | 0.38 ± 0.48a | 0.43 ± 0.17a |
| SOM (g·kg^-1^) | 10.38 ± 0.57c | 29.49 ± 0.33a | 17.65 ± 0.45b |
| $\text{N}\text{O}_{\text{3}}^{\text{-}}\text{-N}$ (mg·kg^-1^) | 1.38 ± 1.17c | 2.58 ± 0.18b | 15.03 ± 0.32a |
| $\text{N}\text{H}_{\text{4}}^{\text{+}}\text{-N}$ | 1.98 ± 0.75c | 20.42 ± 0.40b | 40.72 ± 0.51a |
| AP (mg·kg^-1^) | 0.86 ± 0.06c | 9.50 ± 0.00a | 8.81 ± 0.16b |
| Cd (mg·kg^-1^) | 9.97 ± 0.01a | 0.04 ± 0.01a | 0.06 ± 0.01a |

Note: Data are mean ± standard deviation. Values in the same row with the same letter(s) are not significantly different per the Duncan analysis at *p*< 0.05 between different treatments. EC, electrical conductivity; SOM, soil organic matter; $\text{N}\text{O}_{\text{3}}^{\text{-}}\text{-N}$, nitrate nitrogen; AP, available phosphorus;$\text{ N}\text{H}_{\text{4}}^{\text{+}}\text{-N}$, ammonium nitrogen. CK, control treatment; PB, poplar bark biochar treatment; TP, thiourea-modified poplar bark biochar treatment. The same below.

**Table S2** **Description of soil physicochemical properties and enzyme activity in the CK, PB and TP treatment groups.**

| Properties | Treatment | | |
| --- | --- | --- | --- |
|  | CK | PB | TP |
| pH | 7.87 ± 0.09 b | 7.92 ± 0.08 ab | 7.97 ± 0.03 a |
| EC (ms·cm^-3^) | 0.24 ± 0.05 b | 0.20 ± 0.03 b | 0.35 ± 0.13 a |
| SOM (g·kg^-1^) | 6.50 ± 1.48 b | 10.16 ± 2.58 a | 7.07 ± 1.11 b |
| AP (mg·kg^-1^) | 2.68 ± 1.38 c | 23.08 ± 7.92 a | 15.10 ± 3.64 b |
| $\text{N}\text{O}_{\text{3}}^{\text{-}}\text{-N}$ (mg·kg^-1^) | 1.04 ± 0.63 a | 0.61 ± 0.45 b | 0.45 ± 0.70 c |
| $\text{N}\text{H}_{\text{4}}^{\text{+}}\text{-N}$ (mg·kg^-1^) | 10.65 ± 3.18 c | 13.72 ± 3.57 b | 15.02 ± 2.59 a |
| BG | 33.60 ± 13.90 b | 45.87 ± 22.76 a | 51.82 ± 26.31 a |
| Ure | 0.90 ± 0.14 b | 1.09 ± 0.21 a | 1.39 ± 0.56 a |
| PO | 0.50 ± 0.14 b | 0.71 ± 0.24 a | 0.92 ± 0.25 a |

Note: Data are mean ± standard deviation. Values in the same row with the same letter(s) are not significantly different per the Duncan analysis at *p*< 0.05 between different treatments. BG, β-glucosidase (μg p-nitrophenlo·g^-1^·h^-1^); Ure, urease (mg $\text{N}\text{O}_{\text{3}}^{\text{-}}\text{-N}$·g^-1^·24h^-1^); Phos, alkaline phosphatase (mg phenol·g^-1^·24h^-1^). The same below.

**Table S3 Topological properties of the co-occurrence network**.

| Topological parameters | Treatments | | | | | | | | | | | | | | |
| --- | --- | --- | --- | --- | --- | --- | --- | --- | --- | --- | --- | --- | --- | --- | --- |
|  | CK | | | | PB | | | | | | TP | | | | |
|  | Bacteria | Fungi | Bacteria- Fungi | | Bacteria | | Fungi | | Bacteria- Fungi | | Bacteria | | Fungi | | Bacteria- Fungi |
| R^2^ | 0.835 | 0.843 | 0.907 | 0.879 | | 0.884 | | 0.897 | | 0.915 | | 0.905 | | 0.921 | |
| Nodes | 286 | 128 | 414 | 580 | | 152 | | 554 | | 402 | | 192 | | 772 | |
| Clustering coefficient | 0.71 | 0.78 | 0.88 | 0.89 | | 0.84 | | 0.92 | | 0.92 | | 0.87 | | 0.92 | |
| Average_degree | 22.50 | 11.33 | 28.04 | 31.73 | | 9.83 | | 39.26 | | 51.02 | | 14.94 | | 61.90 | |
| Betweenness_centralization | 0.09 | 0.12 | 0.06 | 0.08 | | 0.21 | | 0.12 | | 0.18 | | 0.28 | | 0.19 | |
| Modularity | 0.72 | 0.70 | 0.74 | 0.74 | | 0.73 | | 0.75 | | 0.76 | | 0.78 | | 0.77 | |

Note: R^2^: the credibility of scale-free networks.

# Supplementary Figures


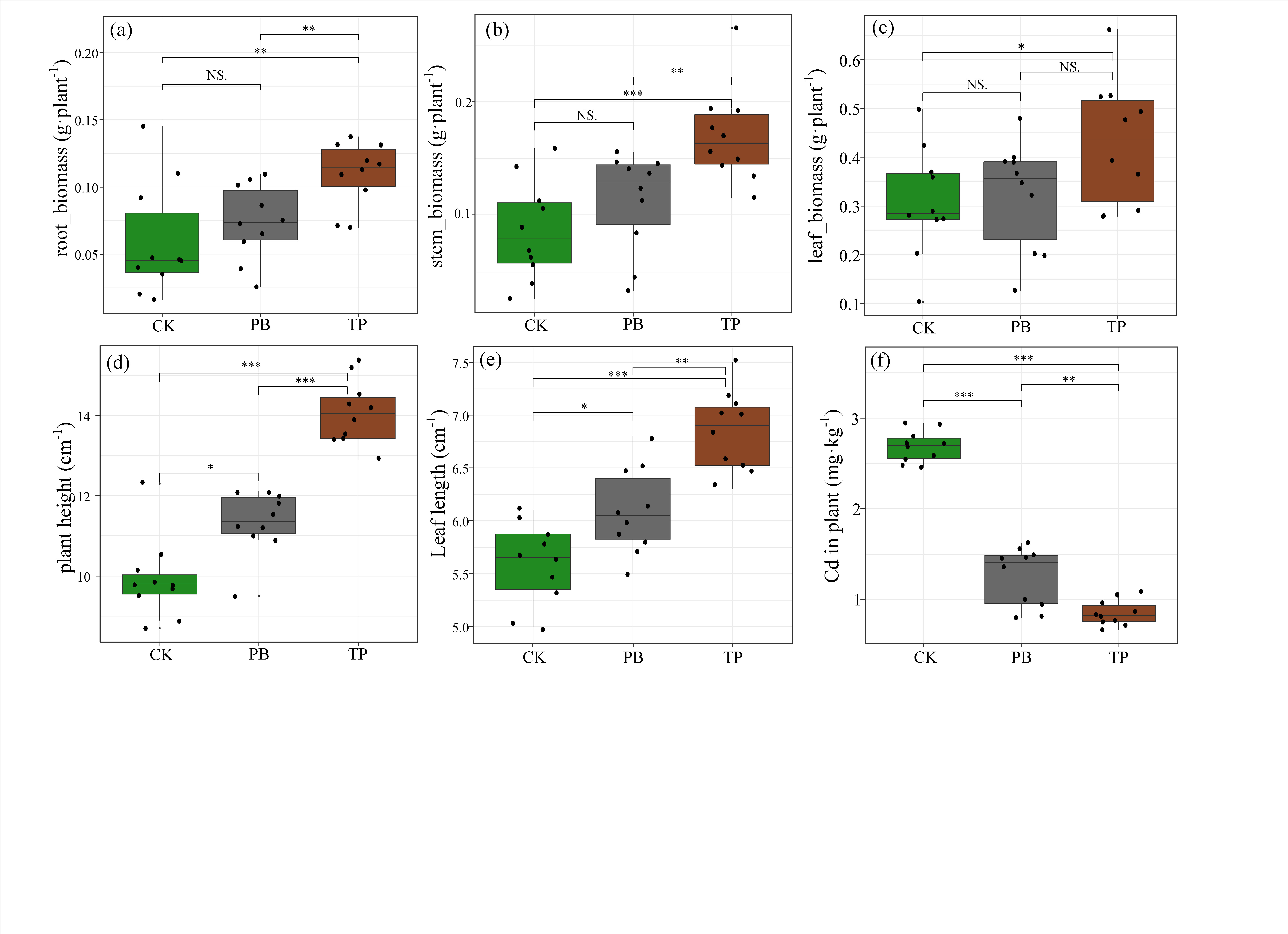


**Supplementary Figure S1. Effect of biochar application on cabbage physiology and Cd content in cabbage.** (a): root biomass, (b): stem biomass, (c): leaf biomass, (d): plant height, (e): leaf length, (f): Cd conent in cabbage. Asterisks indicate the statistical significance (****p* < 0.001, ***p* < 0.01, **p* < 0.05 and NS, *p* > 0.05).

#

**Figure S2. Effect of biochar application on cabbage physiology and Cd content in cabbage.** Relative abundance of abundant (upper left) and rare (upper right) bacterial phyla; and (b) fungal abundant (upper left) and rare (upper right) class in CK, PB and TP treatment.


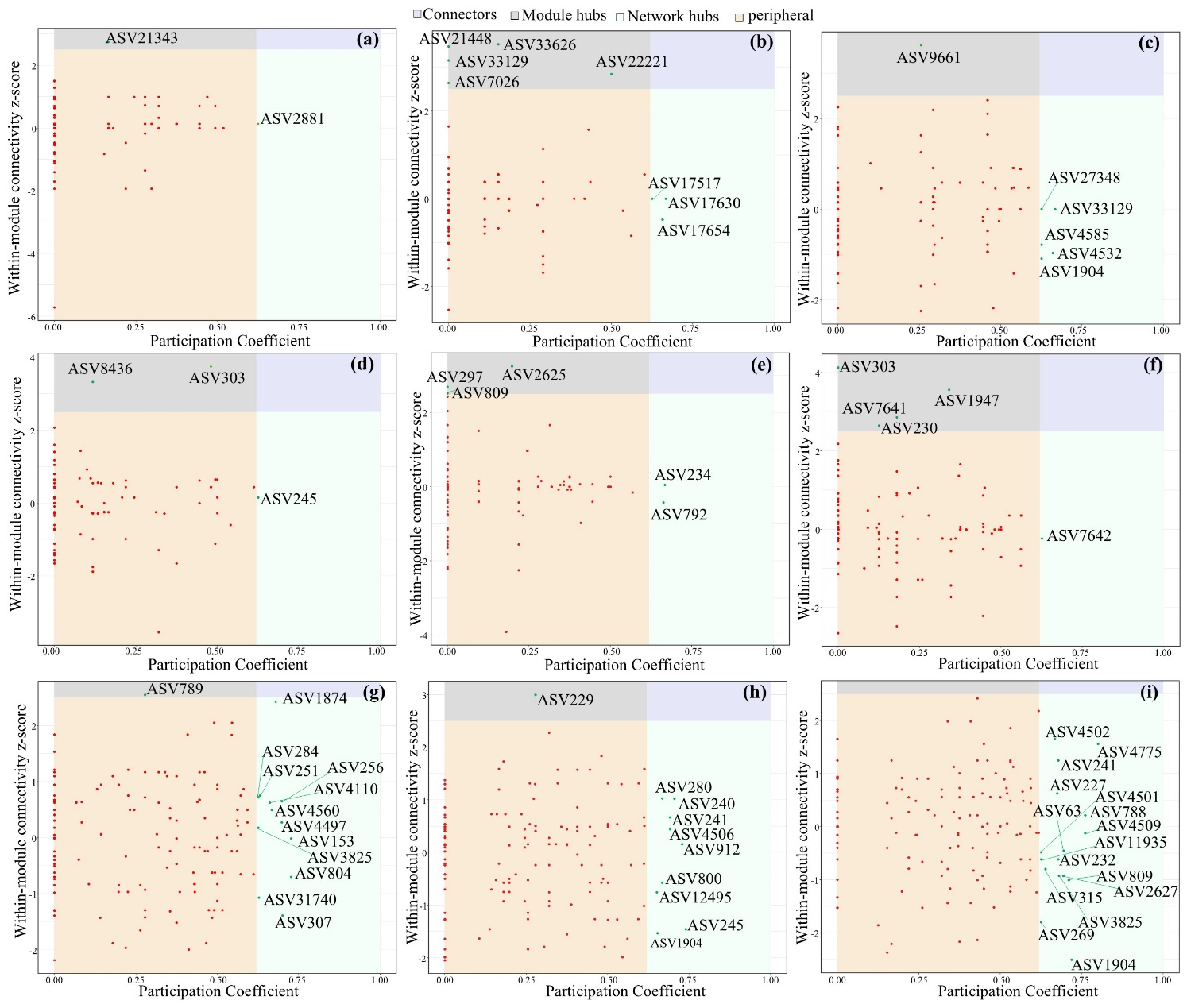


**Figure S3.** **Z-P plot of co-occurrence networks in CK, PB, and TP treatments.** *Proteobacteria*: ASV7026, ASV1904, ASV17630, ASV4497, ASV4110, ASV4560, ASV1904, 11935, ASV4775, ASV4501, ASV1904, ASV4509; *Actinobacteriota*: ASV2881, ASV33129, ASV33626, ASV33129, ASV3825, ASV4506, ASV3825; *Chloroflexi*: ASV21343, ASV21448, ASV4585, ASV912; *Bacteroidota*: ASV4532, ASV4502; *Myxococcota*: ASV22221, ASV31740; *Gemmatimonadota*: ASV17517, ASV9661; *Patescibacteria*: ASV17654; *Bdellovibrionota*: ASV27348; *Ascomycota*: ASV303, ASV245, ASV297, ASV2625, ASV792, ASV809, ASV230, ASV1947, ASV7642, ASV153, ASV804, ASV789, ASV284, ASV280, ASV240, ASV241, ASV229, ASV227, ASV232, ASV315, ASV63, ASV269, ASV788; *Basidiomycota*: ASV8436, ASV7641, ASV1874; *Chytridiomycota*: ASV800; *Unclassified*: ASV256, ASV251, ASV307, ASV2627.
